# Parthenolide reveals an allosteric mode to inhibit the deISGylation activity of SARS-CoV‑2 papain-like protease: Parthenolide covalently inhibits papain-like protease

**DOI:** 10.3724/abbs.2022092

**Published:** 2022-07-20

**Authors:** Zhihui Zou, Huizhuang Shan, Demeng Sun, Li Xia, Yulong Shi, Jiahui Wan, Aiwu Zhou, Yunzhao Wu, Hanzhang Xu, Hu Lei, Zhijian Xu, Yingli Wu

**Affiliations:** 1 Hongqiao International Institute of Medicine Shanghai Tongren Hospital / Faculty of Basic Medicine Chemical Biology Division of Shanghai Universities E-Institutes Key Laboratory of Cell Differentiation and Apoptosis of the Chinese Ministry of Education Chinese Academy of Medical Sciences Research Unit 2019RU043 Shanghai Jiao Tong University School of Medicine (SJTU-SM) Shanghai 200025 China; 2 Laboratory Medicine Guangdong Provincial People’s Hospital Guangdong Academy of Medical Sciences Guangzhou Guangdong 510000 China; 3 Tsinghua-Peking Center for Life Sciences Key Laboratory of Bioorganic Phosphorus Chemistry and Chemical Biology (Ministry of Education) Beijing Advanced Innovation Center for Structural Biology Department of Chemistry Tsinghua University Beijing 100084 China; 4 CAS Key Laboratory of Receptor Research Drug Discovery and Design Center Shanghai Institute of Materia Medica Chinese Academy of Sciences Shanghai 201203 China

**Keywords:** COVID-19, SARS-CoV-2, PLpro, Parthenolide, deISGylation

## Abstract

The coronavirus papain-like protease (PLpro) of severe acute respiratory syndrome coronavirus 2 (SARS-CoV-2) is responsible for viral polypeptide cleavage and the deISGylation of interferon-stimulated gene 15 (ISG15), which enable it to participate in virus replication and host innate immune pathways. Therefore, PLpro is considered an attractive antiviral drug target. Here, we show that parthenolide, a germacrane sesquiterpene lactone, has SARS-CoV-2 PLpro inhibitory activity. Parthenolide covalently binds to Cys-191 or Cys-194 of the PLpro protein, but not the Cys-111 at the PLpro catalytic site. Mutation of Cys-191 or Cys-194 reduces the activity of PLpro. Molecular docking studies show that parthenolide may also form hydrogen bonds with Lys-192, Thr-193, and Gln-231. Furthermore, parthenolide inhibits the deISGylation but not the deubiquitinating activity of PLpro *in vitro*. These results reveal that parthenolide inhibits PLpro activity by allosteric regulation.

## Introduction

The current outbreak of coronavirus disease 2019 (COVID-19) pandemic, caused by severe acute respiratory syndrome coronavirus 2 (SARS-CoV-2), has led to a global public health emergency [ 1, 2] . SARS-CoV-2 belongs to the genus Betacoronavirus and has a genetic similarity of 83% compared with SARS-CoV [3]. Up to June 23, 2022, WHO reported more than 539 million confirmed cases and 6.3 million deaths worldwide (WHO, 2022, https://covid19.who.int). In response to the pandemic, phenomenal progress has been made, particularly in preclinical and clinical trials, including three COVID-19 vaccines developed by Pfizer/BioNTech, Moderna, and Johnson & Johnson, which the FDA in the United States have approved. Meanwhile, a lot of drugs have been introduced in the treatment of COVID-19 patients. However, the largest randomized trial among hospital inpatients organized by WHO so far showed that repurposed FDA-approved drugs, including remdesivir, hydroxychloroquine, lopinavir, and interferon regimens, appear to have little or no effect on hospitalized COVID-19 patients [4]. Therefore, it is urgent to find new antiviral therapeutics to combat the virus.

One of the potential drug targets encoded by SARS-CoV-2 is the papain-like protease (PLpro), which functions as an essential cysteine protease. Specifically, SARS-CoV-2 PLpro, as a functional domain of nonstructural protein 3 (nsp3), cleaves the viral replicase protein pp1a at three sites and generates three mature proteins nsp1, nsp2, and nsp3 [5]. SARS-CoV-2 PLpro also has other proteolytic activities, such as removing K48-linked ubiquitin (Ub) and ubiquitin-like protein ISG15 (interferon-stimulated gene 15) modifications from intracellular proteins. In particular, it was reported that SARS-CoV-2 PLpro has high activity in removing ISG15, whereas SARS-CoV PLpro predominantly targets ubiquitin chains *in vitro* [ 6, 7] . Due to its deISGylation function, SARS-CoV-2 PLpro is involved in the generation of free (unconjugated) ISG15, which promotes pro-inflammatory cytokine production from immune cells [ 8, 9] . Thus, PLpro is a viable target for developing drugs against SARS-CoV-2.

Since PLpro is both a cysteine protease and a deubiquitinase, we screened several DUB inhibitors and found that parthenolide, a natural product that inhibits USP7 [10], could also inhibit the deISGylation activity of SARS-CoV-2 PLpro via direct interaction. Furthermore, we found that parthenolide covalently binds to Cys-191 or Cys-194 of the SARS-CoV-2 PLpro protein. Our work shows that parthenolide is a novel allosteric inhibitor of PLpro, and is thus a promising lead compound for developing new drugs for SARS-CoV-2 treatment.

## Materials and Methods

### Cell culture and reagents

The human cell lines HeLa and HEK293T were purchased from the American Type Culture Collection (ATCC, Manassas, USA). HeLa and HEK293T cells were cultured in Dulbecco’s modified Eagle’s medium (DMEM; HyClone, Logan, USA) containing 10% fetal bovine serum (FBS; Gibco, Carlsbad, USA) and 1% penicillin-streptomycin (Gibco). All the cells were kept in a 37°C incubator with 5% CO _2_.

Parthenolide was purchased from Sigma-Aldrich (St Louis, USA). GRL0617 was purchased from Targetmol Co., Ltd. (Shanghai, China). Recombinant human ISG15 protein was purchased from SinoBiological (Beijing, China). Recombinant human ISG15-7-amino-4-methylcourmarin (ISG15-AMC) was purchased from R&D Systems (Minneapolis, USA). Recombinant human Ubiquitin-7-amino-4-methylcourmarin (Ubiquitin-AMC) was purchased from Hefei KS-V Peptide Biological Technology Co., Ltd (Hefei, China). K48-linked tri-ubiquitin was purchased from Biovision (Milpitas, USA). Unless otherwise specified, all other chemicals were obtained from Selleckchem (Shanghai, China).

### Expression and purification of wild-type and SARS-CoV-2 PLpro mutants

The pGEX6P-1 bacterial expression vector carrying PLpro was kindly provided by Prof. Lei Liu (Tsinghua University, Beijing, China). PLpro C191S, C194S and C191S/C194S mutants were generated from wild-type PLpro plasmid using Mut Express II Fast Mutagenesis Kit V2 (Vazyme Biotech, Nanjing, China) and verified by DNA sequencing. The BL21(DE3) *E. coli* competent cells (Sangon Biotech, Shanghai, China) were transformed with the plasmid, cultured in 2×YT medium at 37°C until the OD _600_ reached 0.6. Then, protein expression was induced by the addition of 0.5 mM IPTG and overnight incubation at 18°C. The cells were harvested by centrifugation, resuspended in the lysis buffer supplemented with protease inhibitor cocktail (Sangon Biotech) and lysed by sonication. After centrifugation at 20,000 *g* for 1 h at 4°C, the supernatant was applied to a GSTrap FF column (5 mL; GE Healthcare, Sunnyvale, USA) and washed with the washing buffer. GST-tagged PLpro proteins were eluted with the elution buffer. PreScission Protease (Beyotime, Shanghai, China) was added to the eluate to remove the GST-tag, followed by dialysis overnight at 4°C. PreScission Protease and GST tags were removed by GSTrap FF column, after which purified wild-type PLpro or its mutants were quantified by SDS-PAGE.

### Plasmids, overexpression and transfection

For mammalian expression, SARS-CoV-2 PLpro was cloned into a pLVX vector (Testobio, Ningbo, China) containing a Flag tag at the N-terminal region. Lentiviral particles were produced in HEK293T cells by co-transfection of packaging plasmid psPAX2 and envelope plasmid pMD2G, using the Lipofectamine 3000 (Invitrogen, Carlsbad, USA) following the manufacturer’s instructions. After 48 h, HeLa cells were infected with viral supernatants and culture medium containing 8 μg/ml polybrene (Sigma-Aldrich).

### Thermal shift assay and cellular thermal shift assay

The thermal shift assay (TSA) and cellular thermal shift assay (CETSA) were conducted as previously described [ 11, 12] . Briefly, purified wild-type or PLpro mutants, or cell lysates from HeLa cells transfected with Flag-tagged SARS-CoV-2 PLpro, were incubated in a final volume of 30 μL in the presence or absence of 100 μM parthenolide (DMSO as a control) for 30 min and heated at different temperatures for 3 min. For the dose-response analysis, equal amounts of the purified protein or cell lysates were incubated with DMSO or different concentrations of parthenolide for 30 min, followed by heating at 53°C for 3 min. For the time-response analysis, equal amounts of the purified protein were incubated with DMSO or different concentrations of parthenolide for 5–30 min, followed by heating at 53°C for 3 min. All heated samples were centrifuged at 20,000 *g* for 20 min at 4°C. The supernatants were subjected to SDS-PAGE and Coomassie Brilliant Blue staining (purified protein) or western blot (cell lysates) analysis.

### Western blot analysis

Equal quantities of protein extracts from HeLa cells were subjected to SDS-PAGE gels (12%) and transferred to nitrocellulose membranes (Millipore, Danvers, USA). After being blocked with 5% defatted milk, the membranes were incubated with primary antibodies overnight at 4°C, followed by incubation with the horseradish peroxidase (HRP)-conjugated secondary antibody (Millipore). The signals were detected using the ECL detection system (Thermo Fisher Scientific, Waltham, USA). Antibodies against Flag (#14793), ISG15 (#2743), Ubiquitin (#9502), and β-actin (#5125) were purchased from Cell Signaling Technology (Dallas, USA).

### Mass-spectrometry (MS)

For mass spectrometric experiments, the mixed protein lysates were reduced for 1 h at room temperature by 10 mM dithiothreitol, then alkylated for 1 h at room temperature in the dark by 55 mM iodoacetamide. The protein was then digested with sequencing-grade modified trypsin (Promega, Madison, USA) with a protein-to-enzyme ratio of 50:1 at 37°C overnight. The tryptic peptides were treated with 1% trifluoroacetic acid, then purified using C18 Ziptips and eluted with 0.1% trifluoroacetic acid in ~50%–70% acetonitrile. The eluted peptides were lyophilized using a SpeedVac (ThermoSavant, Waltham, USA) and resuspended in 10 μL solution containing 1 % formic acid and 5% acetonitrile. All mass spectrometric experiments were performed on an Orbitrap Fusion LUMOS mass spectrometer (Thermo Fisher Scientific) connected to an Easy-nLC 1200 via an Easy Spray (Thermo Fisher Scientific).

All MS/MS ion spectra were analyzed using PEAKS 10.0 (Bioinformatics Solutions Inc, Ontario, Canada) for data processing, *de novo* sequencing, and database searching. The resulting sequences were searched in the UniProt Human Proteome database (downloaded 5th May 2018), with mass error tolerances set to 10 ppm and 0.02 Da for the parent and fragment, respectively. The digestion enzyme semi-Trypsin allowed for two missed tryptic cleavages. The carbamidomethylation of cysteine was specified as a fixed modification, while the oxidation of methionine, acetylation of the N terminus, and parthenolide modification (m/z=248.14) of cysteine were set as variable modifications. FDR estimation was enabled. Peptides were filtered for −log10[P] ≥ 20, whereas proteins were filtered for −log10[P] ≥ 15 and one unique peptide. Proteins sharing significant peptide evidence were grouped into clusters.

### Molecular docking

Molecular docking calculations were performed using the crystal structure of SARS CoV-2 PLpro in complex with peptide inhibitor VIR251 (PDB ID: 6WX4) and prepared with the Protein Preparation Wizard in Maestro module (Schrödinger, LLC, New York, USA, 2020). The parthenolide compound was processed by the LigPrep module (Schrödinger, LLC) for protonation and energy minimization. Subsequently, parthenolide was covalently docked to SARS-CoV-2 PLpro on Cys-191 and Cys-194 by CovalentDock [13] with the default parameters.

### ISG15-AMC/Ubiquitin-AMC assay

The ISG15-AMC or Ubiquitin-AMC assays were performed using the method described previously [7]. Briefly, ISG15-AMC or Ubiquitin-AMC was used as the substrate of PLpro, and the release of AMC was measured by the increase in fluorescence intensity. Purified PLpro (100 nM) was pre-incubated with DMSO or parthenolide (0–400 μM) in the AMC reaction buffer (100 mM NaCl, 50 mM HEPES, pH=7.4) at 37°C for 30 min, after which the mixture was aliquoted into a 384-well plate. The enzymatic reaction was initiated by adding the ISG15-AMC substrate (10 μM) or Ubiquitin-AMC (1 μM) to the well. Fluorescence was measured at 30 s intervals using a microplate reader (Perkin-Elmer, Waltham, USA) (Ex./Em. 380/460 nm). Initial velocities of AMC-release were normalized against the DMSO control. The experiment was repeated three times.

### PLpro activity assay with precursor of ISG15 (proISG15) substrates or K48-linked tri-ubiquitin substrates

To test the effect of parthenolide inhibition, enzyme (100 nM PLpro) and parthenolide (0–200 μM) were incubated in the assay buffer (100 mM NaCl, 5 mM HEPES, pH=7.4) at 37°C for 30 min. To evaluate the cleavage efficiency of enzyme, wild-type PLpro or PLpro mutants were diluted in the assay buffer (100 mM NaCl, 5 mM HEPES, pH=7.4). The reactions were initiated by addition of 10 μM of proISG15 or K48-linked tri-ubiquitin substrate and further incubated at 37°C for certain time, after which the reactions were quenched in 2× SDS-PAGE sample buffer, followed by heating at 98°C for 5 min. The proteins were analyzed by western blot analysis and quantified by Image J software. The PLpro activity (cleavage efficiency against proISG15) were calculated by [cleaved proISG15]/[proISG15].

## Results

### Identification of parthenolide as a novel SARS-CoV-2 PLpro interactor

**Figure 1:**
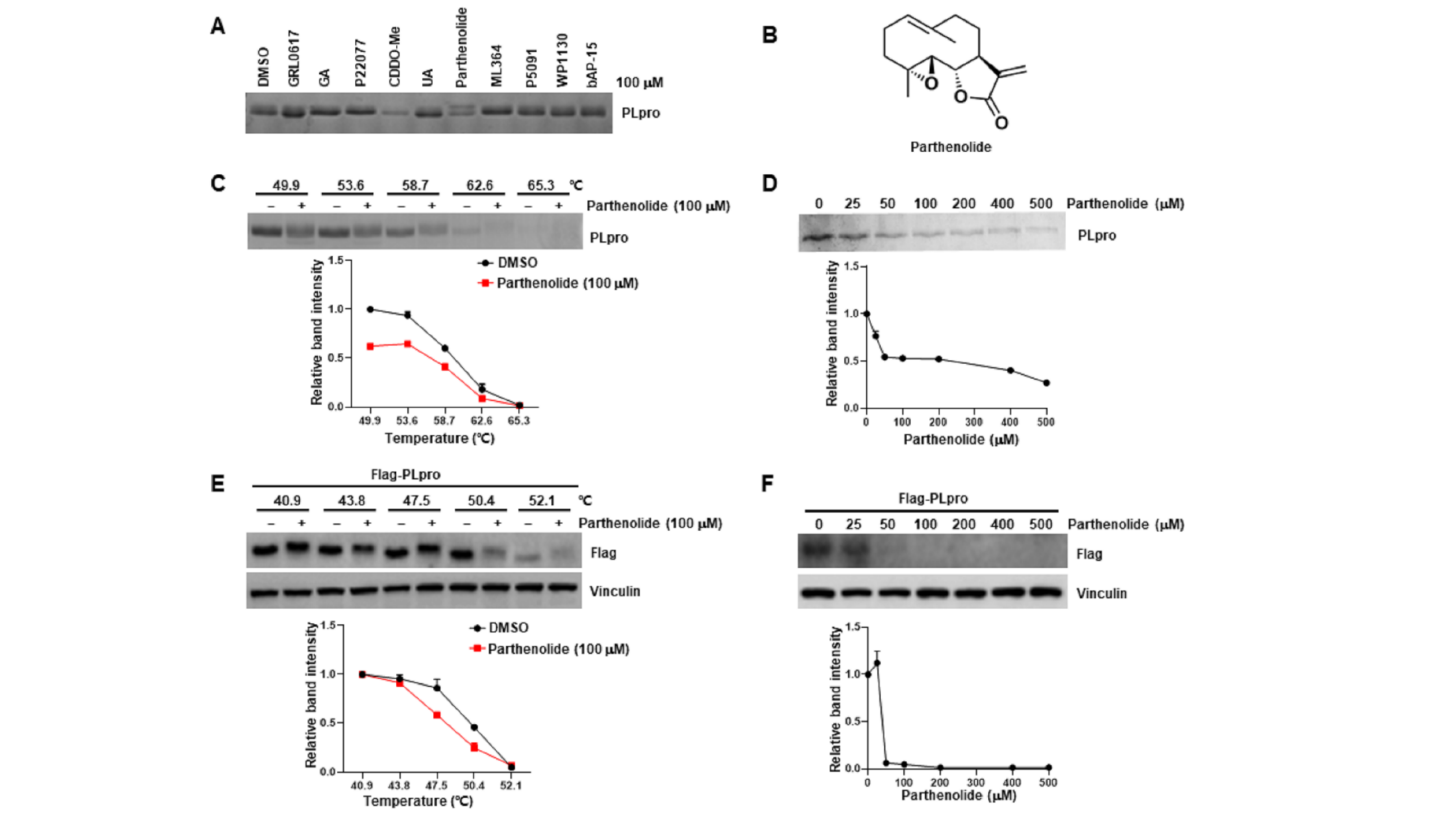
Figure 1 Parthenolide interacts with SARS-CoV-2 PLpro (A) Purified SARS-CoV-2 PLpro protein was incubated with different compounds for 30 min, and the mixtures were subjected to TSA analysis and coomassie brilliant blue staining. (B) The chemical structure of parthenolide. (C,D) TSA was performed on purified SARS-CoV-2 PLpro protein as described in the Materials and Methods section. The effects of parthenolide on PLpro at different temperatures (C) and different doses (D) were evaluated by coomassie brilliant blue staining. The intensity of the PLpro bands was quantified by Image J software. (E,F) CETSA was performed on HeLa cells transfected with Flag-tagged SARS-CoV-2 PLpro as described in the Materials and Methods section. The effects of parthenolide on PLpro at different temperatures (E) and different doses (F) were evaluated by western blot analysis. The intensity of the Flag-tagged PLpro bands was quantified by Image J software.

To identify potential inhibitors of SARS-CoV-2 PLpro, we incubated nine DUB inhibitors and one reported SARS-CoV-2 PLpro inhibitor GRL0617 [7] with purified PLpro protein *in vitro* and performed TSA, an assay that examines the interaction between small molecules and proteins by evaluating the thermal stability of the protein. This screening identified that parthenolide ( Figure 1B) dramatically changed the thermal stability of PLpro protein and resulted in an obvious upshift band as revealed by coomassie brilliant blue staining ( Figure 1A). Furthermore, as shown in Figure 1C, parthenolide significantly decreased the thermal stability of PLpro protein at different temperatures (ΔTm=2.67ºC). The thermal stability of PLpro was also decreased by parthenolide in a dose-dependent manner ( Figure 1D). In addition, to test whether parthenolide interacts with PLpro in cells, CETSA was employed in the lysates of HeLa cells which were stably transfected with Flag-tagged SARS-CoV-2 PLpro. As expected, parthenolide treatment markedly decreased the thermal stability of Flag-tagged PLpro protein at different temperatures compared with the control group ( Figure 1E). Similarly, the thermal stability of Flag-tagged PLpro was tapered by parthenolide in a dose-dependent manner ( Figure 1F). These findings suggest that parthenolide interacts with SARS-CoV-2 PLpro *in vitro* and in cells.

### Determination of parthenolide binding sites in SARS-CoV-2 PLpro

**Figure 2:**
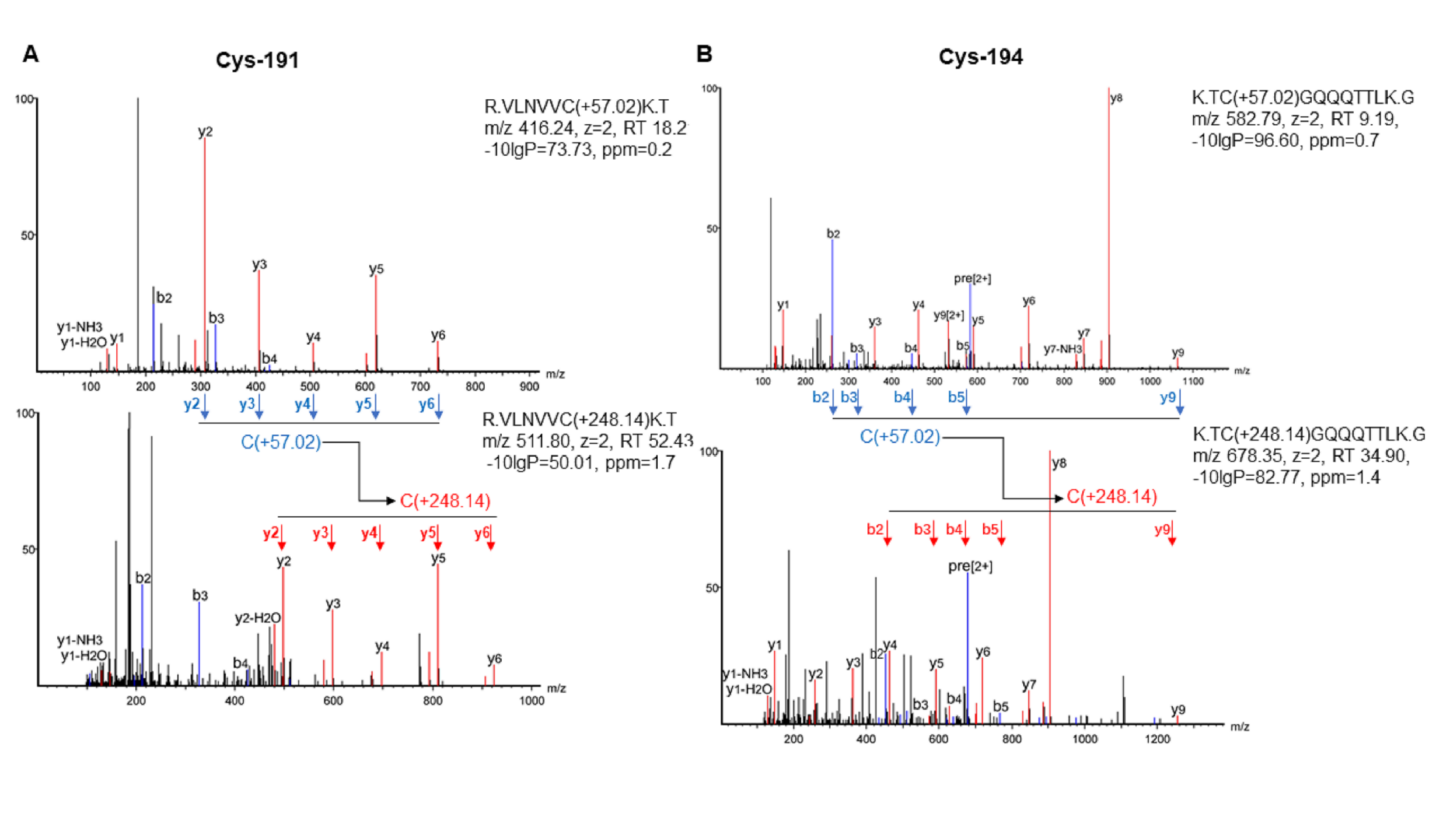
Figure 2 Identification of parthenolide binding sites within SARS-CoV-2 PLpro (A,B) LC-MS/MS proteomics analysis of the Cys-191 (A) or Cys-194 (B) containing tryptic peptide for purified SARS-CoV-2 PLpro protein incubated with (bottom) or without (top) parthenolide for 30 min.

**Figure 3:**
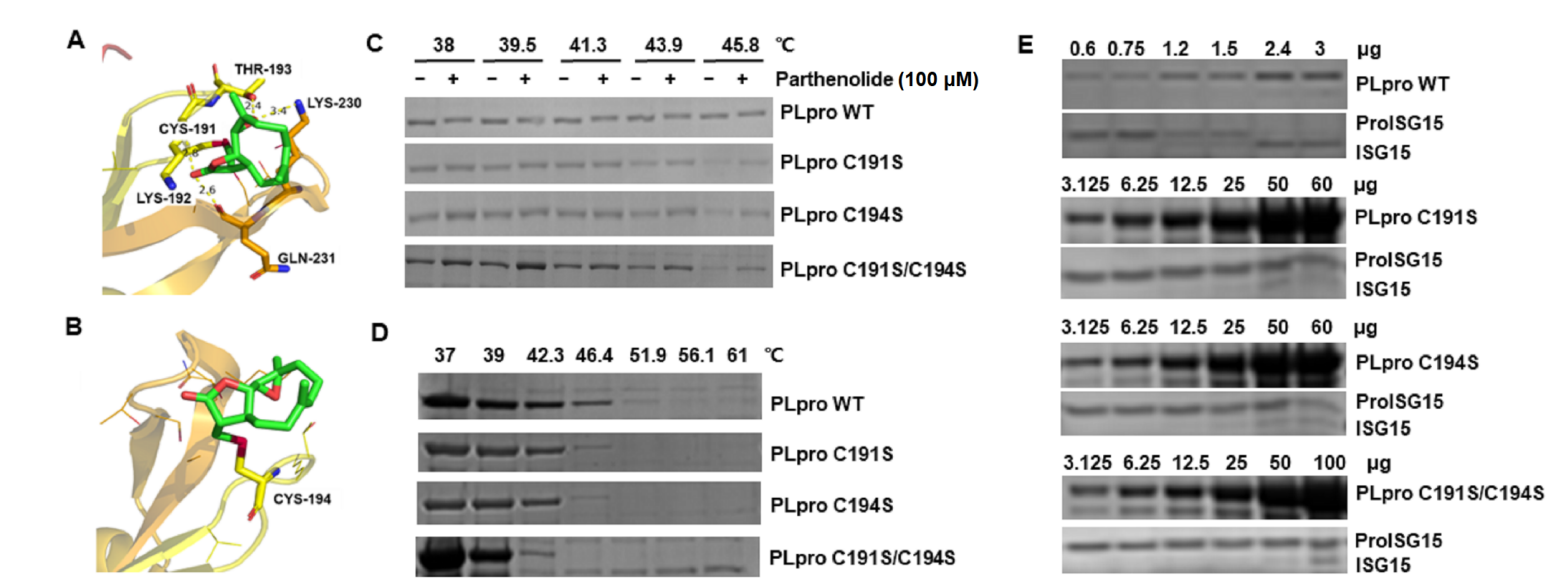
Figure 3 Molecular docking of parthenolide against SARS-CoV-2 PLpro (A,B) Parthenolide covalently binds to SARS-CoV-2 PLpro Cys-191 (A) or Cys-194 (B) in the docking modes. Parthenolide is shown as green sticks and PLpro as ribbons. The essential residues forming hydrogen bonds (yellow dashes) with parthenolide are shown as orange or yellow sticks. The hydrogen bond lengths are labeled in Å. (C) TSA was performed on purified SARS-CoV-2 wild-type PLpro and PLpro mutants in the presence or absence of parthenolide at different temperatures. (D) TSA of purified SARS-CoV-2 wild-type PLpro and PLpro mutants as described in the Material and Methods section. (E) The protease activity against ProISG15 of SARS-CoV-2 wild-type PLpro and PLpro mutants at indicated protein amounts were evaluated as described in the Materials and Methods section.

One interesting finding is that incubation of parthenolide with SARS-CoV-2 PLpro leads to an obvious band shift of PLpro ( Figure 1A,E), indicating that parthenolide may covalently bind to PLpro. Given that parthenolide can covalently bind to the cysteine residuals of some proteins [14], we determined the cysteine(s) in SARS-CoV-2 PLpro possibly targeted by parthenolide. The data in MS showed that two peptides containing cysteines were identified with a mass shift of 248.14 Da, which matched the molecular weight of parthenolide. The identified peptides suggested that parthenolide covalently modified Cys-191 or Cys-194 of PLpro ( Figure 2A,B). Molecular docking showed that parthenolide covalently binds to Cys-191 with a docking score of -7.50 kcal/mol. Molecular docking also showed that parthenolide forms hydrogen bonds with Lys-192, Thr-193 and Gln-231 ( Figure 3A). In another docking mode, parthenolide covalently binds to Cys-194 with a docking score of -7.38 kcal/mol ( Figure 3B). To confirm the MS and molecular docking results, we constructed and purified PLpro C191S, PLpro C194S, and PLpro C191S/C194S proteins. Indeed, the TSA results showed that parthenolide could not induce a band shift for PLpro mutants, which can be observed in wild-type PLpro ( Figure 3C). However, parthenolide could increase the thermal stability of PLpro mutants at different temperatures ( Figure 3C, and Supplementary Figure S1). Interestingly, compared to the wild-type PLpro, the PLpro C191S, PLpro C194S, and PLpro C191S/C194S mutations strongly decreased the thermal stability of PLpro ( Figure 3D). Furthermore, the activity of PLpro for cleavage of proISG15 was markedly declined in PLpro mutants ( Figure 3E, and Supplementary Figure S2), especially in PLpro C191S/C194S, indicating the critical role of Cys-191 and Cys-194 for the activity of PLpro. Overall, these results demonstrate that parthenolide covalently binds to Cys-191 or Cys-194 of PLpro protein and impairs the activity of PLpro.

### Parthenolide inhibits the deISGylation activity of SARS-CoV‑2 PLpro

**Figure 4:**
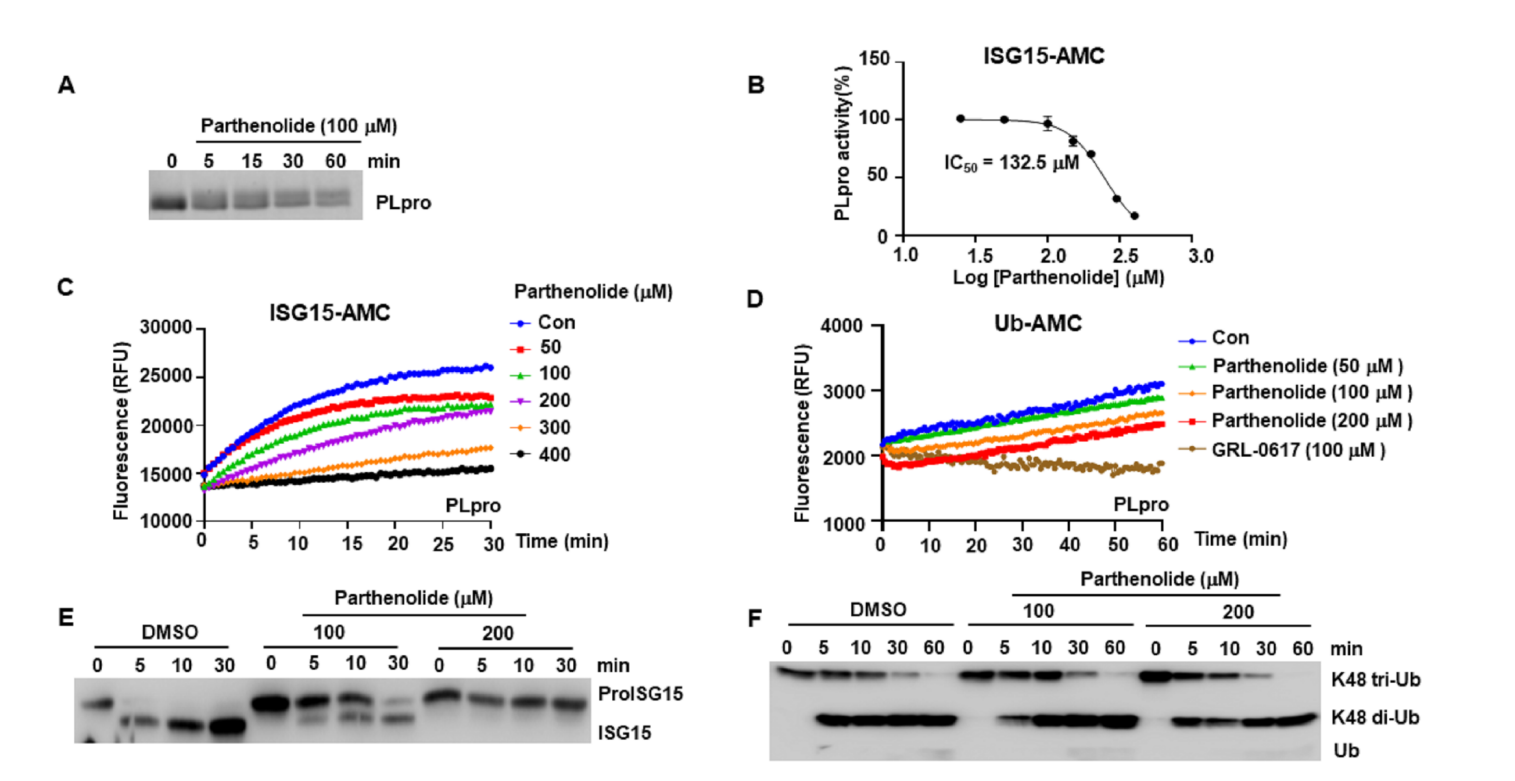
Figure 4 Effect of Parthenolide on SARS-CoV-2 PLpro (A) TSA of purified SARS-CoV-2 PLpro protein treated with parthenolide for different time. (B) Dose-dependent inhibition of SARS-CoV-2 PLpro activity by parthenolide using ISG15-AMC as the substrate. The results are presented as the mean ± SD of three independent experiments. (C) Parthenolide inhibited SARS-CoV-2 PLpro deISGylation activity in concentration- and time-dependent manners in the ISG15-AMC assay. (D) Parthenolide could not inhibit SARS-CoV-2 PLpro deubiquitinating activity in the Ub-AMC assay. (E) SARS-CoV-2 PLpro protein was incubated with parthenolide for 30 min. Then, proISG15 was added and the mixture was incubated for indicated time, followed by western blot analysis with anti-ISG15 antibody. (F) SARS-CoV-2 PLpro protein was incubated with parthenolide for 30 min. K48-linked tri-ubiquitin was then added and incubated for indicated time, followed by western blot analysis with anti-ubiquitin antibody.

To evaluate the time needed to form a covalent bond between parthenolide and SARS-CoV-2 PLpro, we incubated purified PLpro protein with parthenolide for different time (5, 15, 30, and 60 min). The mixtures were subjected to SDS-PAGE and the gel was stained by coomassie brilliant blue. The results showed that parthenolide could modify PLpro as early as 5 min, and with incubation time prolonged, the amount of the upper bands (represents the parthenolide modified PLpro) increased, while the amount of the lower bands (represents un-modified PLpro) decreased ( Figure 4A). These results indicate that parthenolide binds to PLpro in a time-dependent manner.

We then assessed the effect of parthenolide on SARS-CoV‑2 PLpro activity. PLpro was reported to preferentially remove ubiquitin-like ISG15 protein modifications rather than ubiquitin [ 6, 7] . The ISG15-AMC assay results revealed that parthenolide inhibited PLpro deISGylation activity in a dose- and time-dependent manner ( Figure 4B,C). To further confirm this observation, purified PLpro protein was incubated with parthenolide or DMSO, followed by the addition of the precursor of ISG15 (proISG15) substrates. As shown in Figure 4E, compared with the DMSO-treated group, the ability of SARS-CoV-2 PLpro to cleave the proISG15 was blocked by parthenolide in a time- and dose-dependent manner. Intriguingly, parthenolide could not inhibit the deubiquitinating activity of PLpro when Ub-AMC or K48-linked tri-ubiquitin is used as the substrate ( Figure 4D,F). However, parthenolide could inhibit the deubiquitinating activity of USP7 ( Supplementary Figure S3), as reported previously [10]. These data suggest that parthenolide inhibits the deISGylation activity of SARS-CoV‑2 PLpro.

## Discussion

Because of the uncertain outcome of several tested drugs, such as hydroxychloroquine and remdesivir, developing novel anti-COVID-19 drugs is urgently required. Herein, we report that parthenolide is a novel covalent SARS-CoV-2 PLpro inhibitor, implying its potential in the treatment of COVID-19 and for the development of novel PLpro inhibitors.

Parthenolide is a promising candidate for the treatment of COVID-19. Parthenolide and its derivative DMAPT have been used in the phase I trial to treat several inflammatory diseases. Mechanistic investigation showed that parthenolide could inhibit the NF-κB signaling pathways by directly targeting IKKβ or p65 [15]. Parthenolide could also significantly reduce IL-1, IL-2, IL-6, IL-8, and TNF-α production in several human cell line models *in vitro* [ 16, 17] . Based on these data, Bahrami *et al*. [18] proposed that parthenolide may be used to reduce the “cytokines storms” in COVID-19 patients. Interestingly, it has also been reported that inhibiting PLpro may decrease the “cytokine storms” associated with COVID-19 [9]. In the present study, we demonstrate that parthenolide is a direct SARS-CoV-2 PLpro inhibitor, which is supported by the following evidence: firstly, parthenolide could directly bind to PLpro *in vitro* and in cells, as shown by the cellular thermal shift assay; secondly, parthenolide could inhibit the deISGylation activity of PLpro *in vitro* . Thus, in principle, parthenolide has a potential clinical value in the treatment of COVID-19.

Up to date, there are several kinds of PLpro inhibitors, including covalent and non-covalent inhibitors [ 19– 26] . Naphthalene inhibitors, however, are the only compounds that have been extensively studied and subject to lead optimization. Naphthalene derivatives, such as GRL0617, inhibits the PLpro activity by non-covalently occupying the substrate pockets and sealing the entrance to the substrate-binding cleft [ 7, 27] . Unfortunately, no naphthalene derivatives have entered the clinical trial. Unlike naphthalene derivatives, parthenolide is a novel covalent inhibitor of PLpro. Parthenolide has been reported as a covalent inhibitor for IKKβ [15] and p65 [28]. From a structural point of view, we compared the inhibitory sites of parthenolide between its known targets IKKβ, p65, and PLpro, and used CovalentDock to dock the parthenolide with these proteins ( Supplementary Figure S4). According to the docking binding modes, non-bonded interacting amino acids in p65, IKKβ, and PLpro did not show a high degree of overlap. However, it was observed from the structure of the inhibitory site that the cysteines are all located on the flexible protein loop, and the binding pockets are narrow and close to the solvent zone, indicating that only compounds with small molecular weights can be accommodated. In addition, the covalent reaction of cysteine further enhanced the binding of the parthenolide to these three targets. Compared with the reported non-covalent inhibitors for PLpro, the covalent binding character of parthenolide indicates that even a short exposure may cause the permanent loss of PLpro activity without the need for high concentration or long-term treatment. Interestingly, Cys-191 or Cys-194, instead of Cys-111 at the PLpro catalytic site [29], are targeted by parthenolide, indicating a potential allosteric site for inhibiting PLpro activity. More importantly, the proISG15 cleavage assay showed that mutations of Cys-191 or/and Cys-194 of PLpro markedly decreased the deISGylation activity of PLpro ( Figure 3E, and Supplementary Figure S2), indicating the importance of these two cysteines in maintaining the activity of PLpro. Another interesting finding is that parthenolide inhibits the deubiquitinating activity of USP7, but it inhibits the deISGylation activity of PLpro instead of its deubiquitinating activity ( Supplementary Figure S3). Whether parthenolide binds to PLpro closer to its S1 site, which prefers ISG15 modifications, or S2 site, which prefers K48-polyubiquitin, remains to be resolved [6].

Considering the rapid spread of COVID-19 and the urgent requirement for agents against COVID-19, our data suggest that parthenolide may serve as the basis for developing new PLpro inhibitors. It has been reported that parthenolide exhibits cytotoxicity in HEK-293T at concentrations 29.9 μM [30]. We found that even at 60 μM ( Supplementary Figure S5), parthenolide did not induce cell death in Vero-E6 cells. Thus, parthenolide might be used at a relatively higher concentration. In addition, due to the covalent binding nature of parthenolide, low concentrations of parthenolide are expected to cause consistent loss of PLpro activity [31]. Nevertheless, there are a few limitations in this work. For example, we did not examine the effect of parthenolide on SARS-CoV-2-infected cells due to the lack of P3 experimental facilities.

In conclusion, we demonstrate that parthenolide is a potential lead compound for developing PLpro inhibitors. Since parthenolide has been clinically used, our data may provide the basis for the clinical evaluation of parthenolide in the treatment of COVID-19.

## Supplementary Data

Supplementary data is available at *Acta Biochimica et Biophysica Sinica* online.
